# Perceptions and Practices of Interdisciplinary Action in an Intra-Hospital Support Team for Palliative Care: A Qualitative Study

**DOI:** 10.3390/healthcare13101179

**Published:** 2025-05-19

**Authors:** Célio Cruz, Ana Querido, Vanda Varela Pedrosa

**Affiliations:** 1 School of Health Sciences of the Polytechnic University of Leiria, Campus 2, Morro do Lena, Alto do Vieiro, Apartado 4137, 2411-901 Leiria, Portugal; cegocr@gmail.com (C.C.); ana.querido@ipleiria.pt (A.Q.); 2 Center for Innovative Care and Health Technology (ciTechCare), Polytechnic University of Leiria, Campus 5, Rua das Olhalvas, 2414-016 Leiria, Portugal

**Keywords:** palliative care, interdisciplinarity, team

## Abstract

**Background:** The quality of palliative care (PC) services is closely linked to the effectiveness of interdisciplinary collaboration. A coordinated approach among professionals from different fields fosters holistic, person-centered care, ensuring comprehensive support for patients with complex conditions and their families. In hospital settings, In-Hospital Palliative Care Support Teams (EIHSCPs) play a key role in delivering specialized care, enhancing interdepartmental communication, training other healthcare professionals, and optimizing resources. Strong leadership by PC specialists, combined with effective team management, contributes to symptom relief, improved quality of life, and cost reduction. However, interdisciplinary collaboration presents challenges, including competing priorities, resource constraints, and communication barriers. Despite its recognized benefits, research on its implementation in PC, particularly in Portugal, remains scarce. **Objective:** This study explores the perspectives and practices of professionals within an EIHSCP, examining team dynamics, interprofessional collaboration, and key facilitators and barriers. **Methods**: Twelve semi-structured interviews were conducted with physicians, nurses, psychologists, and social workers from the EIHSCP in the Médio Tejo region. Data were analyzed using Braun and Clarke’s reflexive thematic analysis. **Results**: The interview findings were organized into three themes: (1) Social Representations and Interdisciplinary Practice; (2) Competencies for Interdisciplinary Practice; and (3) Challenges in Interdisciplinary Practice. Participants consistently highlighted that interdisciplinary collaboration enhances communication between services and improves care quality. While teamwork is central, patient- and family-centered care remains the priority. Key competencies include empathy, ethics, active listening, and cultural sensitivity, alongside structural and procedural elements such as team meetings, integrated communication, and clear referral criteria. Continuous education and professional development are essential. Challenges primarily stem from limited human and material resources, staff workload and stress, communication gaps between hospital and community teams, and insufficient institutional recognition. Suggested improvements focus on investing in ongoing training, strengthening communication and inter-institutional collaboration, and revising the organizational model of PC within Portugal’s National Health Service. **Conclusions**: Interdisciplinary collaboration in PC is fundamental for holistic, patient-centered care but is hindered by structural and organizational barriers.

## 1. Introduction

A united and well-prepared team working toward a common goal in healthcare delivery can be highly effective in overcoming challenges. The interdisciplinary approach in healthcare enhances process efficiency and dynamism through collaborative expertise; however, it also introduces a level of complexity that requires effective management [1,2,3].

Interdisciplinary collaboration offers countless advantages in addressing contemporary challenges, achieving objectives, and improving health outcomes. It brings together professionals not only from the healthcare sector but also from social and other relevant fields, fostering expertise in specific professional domains while also enhancing interdisciplinary practice. The challenges faced by teams in these settings are inherently different from those encountered by non-healthcare teams [4,5,6,7].

Several facilitating factors contribute to effective interdisciplinary teamwork, including the establishment of a collaborative culture, strong and solid communication, task interdependence, and shared objectives [1,8]. Additionally, various societal factors indicate a growing need for interdisciplinary healthcare teams: (1) an aging population and an increasing number of patients with complex and chronic conditions; (2) the rising complexity of competencies and knowledge required for healthcare delivery; (3) the increasing specialization of healthcare professions, leading to greater fragmentation; (4) policies promoting multiprofessional teams and continuous learning; and (5) the integration of person- and community-centered care [4,5,6,7,8,9,10].

Nevertheless, despite its numerous advantages, multifactorial interdependence can expose teams to conflicting priorities and power dynamics, a lack of recognition for team members, inexperience in collaborative work, member overload due to insufficient resources or expertise, and leadership that fails to foster collaboration, among other challenges that require careful attention [1,4,5,6,8,10,11,12].

Some recurring themes that help address these challenges include recognizing barriers, avoiding the path of least resistance, encouraging and modeling collaboration, supporting shared mental models regarding roles and priorities, fostering a sense of psychological safety, and conducting regular team debriefings [2,11].

Considering this, it is crucial to recognize that healthcare must be coordinated and intersectoral to ensure that individuals in situations of greater vulnerability achieve an improved quality of life and safety at the right time [10,13].

To achieve this, greater emphasis must be placed on the topic and its research, as current knowledge remains limited, both internationally and within the Portuguese context. Notably, the advancement of knowledge on interdisciplinary education has outpaced the development of effective interdisciplinary solutions within healthcare teams [5,6,7,10,14].

Regarding education, the integration of interprofessional competencies into curricula, including workplace-based learning, is essential for consolidating effective interdisciplinary practice [1,7,10,14]. Interdisciplinary education and training contribute to a better understanding of responsibilities, roles, and functions, particularly in the following areas: (1) ensuring appropriate support for individuals transitioning between care settings; (2) encouraging interdisciplinary teamwork; (3) defining each member’s responsibilities in care coordination; (4) strengthening the role of volunteers; and (5) tailoring care to specific needs [2,3,15,16,17,18].

In the context of healthcare, interdisciplinary practices, whether influenced by the range and types of services or the variability in care delivery settings, necessitate a clear definition of roles and responsibilities among team members. This includes recognizing the contributions of all involved, including the patient and their family. It is essential to simplify the understanding and respect of professional boundaries, streamline referral and consultation processes, and ensure a continuous flow of communication [2,3,5,7,8,10,15,19].

Considering this framework, when analyzing palliative care (PC), it becomes evident that these services must inherently be provided by an interdisciplinary team. Such an approach is crucial to meeting the needs of individuals facing life-threatening illnesses and their families, ensuring timely support in alignment with the World Health Organization (WHO) definition of PC, widely recognized as the gold standard across Europe [20,21,22,23,24].

Among the ten core competencies in PC, particular emphasis is placed on their implementation through a fully integrated interdisciplinary coordination model, regardless of the setting in which care is delivered [6,20,25]. According to Twycross [26] and Radbruch et al. [22], these services should be grounded in a set of globally acknowledged values and principles, including autonomy, dignity, the patient–healthcare professional relationship, quality of life, perspectives on life and death, communication, public education, interdisciplinary teamwork, family support, and symptom management. Among these principles, communication stands out as a decisive factor in the implementation of PC and in ensuring the effective functioning of interdisciplinary teams [24,27].

In this context, by analyzing the organizational model for the provision of PC within the National Health System (SNS) in Portugal, it is evident that it is structured around specialized teams, units, and services. This integrated organization is designed to address the multidimensional needs of patients and families while ensuring close coordination with other healthcare services and levels within the SNS [28].

These PC services may be established at different levels of the system, operating within a network-based framework that includes various types of structures: Palliative Care Units, In-Hospital Palliative Care Support Teams (EIHSCPs), and Community Palliative Care Support Teams [2,3,4,13,15,16,17,29,30].

The focus is placed on the EIHSCPs, which are expected to be highly specialized in delivering PC within a hospital setting. These teams are unique in this setting and operate on an interdisciplinary basis, providing care to patients with high levels of complexity and support to their caregivers [2,3,4,12,16,17,20,29].

In addition to direct patient care, these teams also provide training, establish connections with other community services, and offer consultations to professionals across different levels of the SNS. Furthermore, they collaborate with universities, health schools, and research centers [2,3,30,31].

The fact that hospital-based PC is delivered by interdisciplinary teams led by a specialist in palliative medicine appears to enhance symptom relief, improve quality of life, and optimize multiple care indicators for individuals at the end of life and those with highly complex illnesses. Additionally, this approach contributes to reducing healthcare costs [12,13,30,32,33].

In summary, this coordinated action is increasingly crucial in caring for the most vulnerable individuals, those who suffer the most, those who are dying, and children with complex or incurable conditions. On an international level, knowledge about hospital-based PC remains limited, and in the Portuguese context, it is even more scarce [20].

In this context, it is essential to examine, in detail, the proposal by McLaney et al. [34], who highlighted the high complexity of the hospital setting and the significant benefits of an interdisciplinary approach based on core and collective competencies applicable to the entire team. These include communication, the resolution of interprofessional conflicts, shared decision-making, reflection, role clarification, and interprofessional values and ethics.

From another perspective, Nancarrow et al. [3] summarized key competencies that support effective interdisciplinary work focused on outcomes, including the following: leadership and management, communication, personal rewards, training and development, appropriate resources and processes, team composition, team climate, individual characteristics, a clear vision, a focus on quality and care outcomes, and a respect for and understanding of roles.

Simultaneously, within this setting, PC must also consider the balance between patient discharge and hospital bed availability, a crucial factor in both clinical and organizational outcomes [10,35]. In other words, adherence to established ratios and the specific composition of professionals within the EIHSCP in general hospitals (adults) must be observed. According to national legislation and strategic plans, these teams should necessarily include physicians (1.5 FTE, ideally two to three professionals), nurses (2 FTE, ideally two to three professionals), psychologists (0.5 FTE, minimum of two professionals), and social workers (0.5 FTE, minimum of two professionals) [35]. In this context, the intervention is primarily focused on managing immediate symptoms, with the team’s efforts directed toward handling acute and critical conditions [33,36].

Based on the theoretical framework, the objective of this study is to explore the perceptions and practices of PC professionals regarding their interdisciplinary work within an EIHSCP. Specifically, this study aims to describe the meanings attributed to interdisciplinarity, characterize the most commonly used interdisciplinary competencies, and identify the facilitators and barriers that professionals encounter in implementing interdisciplinary care.

## 2. Materials and Methods

### 2.1. Study Design

This study explores the dynamics of interdisciplinary collaboration in PC within a hospital setting, focusing on healthcare professionals’ perspectives on teamwork across different specialties. It aims to identify key facilitators and challenges that shape interdisciplinary practice. To this end, a qualitative descriptive design was employed, and data were collected through individual semi-structured interviews (Appendix A). Data were analyzed using a thematic approach, following a deductive framework [37,38,39]. The research adhered to the Consolidated Criteria for Reporting Qualitative Research (COREQ) guidelines [40].

### 2.2. Study Setting, Participants, and Recruitment

The participants were voluntarily recruited from an inpatient PC unit in the Médio Tejo region, central Portugal, considering the researcher’s proximity to the field. This team provides interdisciplinary PC to patients and their families within a hospital setting. Sampling followed an intentional approach, with inclusion criteria as follows: (1) professionals working in an inpatient PC unit, (2) directly involved in patient and family care, and (3) having signed the informed consent form. Eligibility criteria were assessed by the lead researcher (V.V.P.). No restrictions were placed on age, sex, religious affiliation, or professional seniority, thereby promoting maximum variation within the sample.

All available team members were invited to participate, and as a result, the saturation reflects the entire target population. Since this study focused specifically on this particular team, all professionals directly involved in PC were interviewed. Therefore, the sample fully represents the population under study. A total of 12 professionals, all members of the EIHSCP, participated in this study, with data collection continuing until thematic saturation was reached.

### 2.3. Data Collection

Data collection was conducted through semi-structured interviews between April and June 2024. A pilot interview was carried out initially, leading to minor adjustments in the interview guide, allowing its inclusion in the final dataset. All interviews were conducted in person and audio-recorded. The interviews were conducted by a white male social worker (C.C.) with ten years of professional experience in PC, under the supervision of a faculty member (V.V.P.) with expertise in both PC and qualitative research.

Each participant was interviewed individually in a quiet location of their choice within the hospital unit, at a prearranged date and time. Interview durations were kept flexible to accommodate the schedules of healthcare professionals, thereby fostering more spontaneous and candid responses.

At the start of each interview, participants completed a sociodemographic questionnaire covering age, gender, years of professional experience, years working in PC, and training in PC (advanced or other types). After the interview, participants were thanked and given the opportunity to ask questions. Following each interview, field notes were recorded to document the researcher’s reflections on the conversation.

Interview questions were presented in a neutral manner, allowing participants to express their own perspectives. All interviews were conducted in the participants’ native language, Portuguese. The duration of the interviews ranged from 4 min and 2 s to 11 min and 43 s. The interviews were translated into English and coded using participant numbers (P1, P2, etc.), along with their professional roles. During translation, quotations were first rendered literally and then refined to ensure equivalence in meaning and interpretation. All authors reviewed both the original Portuguese and the English versions of the quotations. Transcripts were returned to participants for feedback or corrections before proceeding with data analysis.

Although some interviews lasted as little as four minutes, the interview guide was structured around key themes to ensure that each response generated relevant and valuable insights. Participants were encouraged to reflect and provide illustrative examples, resulting in coherent and meaningful narratives, even in shorter sessions. All interview questions were addressed by every participant, with no items omitted during data collection.

### 2.4. Ethical Procedures

This study was conducted in accordance with the principles of the Declaration of Helsinki and was approved by the Ethics Committee of the Local Health Unit of Medio Tejo (Order No. 67/PCA/CA from the Board of Directors). All participants provided informed consent, and their identities were protected using an alphanumeric coding system. Participants were informed that they could withdraw from the study at any stage. No financial compensation was provided for participation.

### 2.5. Data Analysis and Trustworthiness

Using Braun and Clarke’s reflexive thematic analysis, the data were examined to establish the conceptual framework of key themes emerging from the interviews [37,38]. Each interview was analyzed individually, with the initial coding process applying sentence-length excerpts from the text. Codes were then categorized into themes and refined after each interview [41]. Theme validity was ensured through a meticulous review of all codes and the complete dataset. Themes were subsequently refined, named, and organized into a coherent thematic hierarchy. The final report was developed with reference to the relevant literature [41].

After five interviews, the coding process evolved to include longer excerpts spanning multiple sentences. The approach transitioned from descriptive to interpretative coding, aiming to uncover broader relationships within participants’ experiences. Following the completion of each interview, the coding matrix underwent a comprehensive review to identify prominent elements in participants’ narratives. Although the number of participants was limited, each theme was supported by multiple accounts from professionals with diverse backgrounds, highlighting the richness of the data. Every emergent category was illustrated with at least two quotations, with particular attention given to including perspectives from different disciplines. This strategy avoided an over-reliance on repeated viewpoints from a single profession and contributed to the depth and triangulation of the findings.

Coding was carried out by two researchers: the first author (C.C.) coded all transcripts, while the lead researcher (V.V.P.), who has expertise in data analysis, independently co-coded them. Discrepancies in coding were resolved through discussion until consensus was reached. In cases where disagreement persisted, a third reviewer (A.Q.) was consulted to make the final decision. The inter-coder agreement was approximately 90%.

To ensure the trustworthiness of the analytic process, C.C. and V.V.P. held regular meetings to deliberate and refine the wording and content of codes, as well as the conceptual relationships among codes, themes, and subthemes. Peer debriefing was employed to enhance the credibility of the analysis. In accordance with the established principles of qualitative research, an audit trail was maintained, comprising field notes, coded transcripts, and annotations or revisions made during group coding sessions. All members of the research team had prior experience in both PC and qualitative research [41].

The qualitative data analysis software MaxQDA (Version 24.8.0_Portugal) was used for data storage and management.

## 3. Results

### 3.1. Sample Description

A total of 12 interviews were conducted with professionals from the EIHSCP of the Local Health Unit of Médio Tejo (Local Health Units (*Unidades Locais de Saúde*)—integrated organizations that coordinate primary, hospital, and community care within a specific geographic area in Portugal). Of these, 9 were women, with a mean age of 34.2 years (ranging from 26 to 47 years).

Regarding professional roles, the sample included six nurses, two physicians, two social workers, and two psychologists. Participants had an average of 14.3 years of professional experience and 9.3 years of work in PC.

In terms of training, eight participants had advanced training in PC, while ten had completed other types of PC education. A detailed sample description is provided in Table 1.

### 3.2. Study Results

The interview data were summarized into three main themes:

The first theme, “Social Representations and Interdisciplinary Practice”, includes three subthemes: team, patient, and family.

The second theme, “Competencies for Interdisciplinary Practice”, is divided into five subthemes: individual characteristics, resources/procedures, personal rewards/training/development, communication, and interprofessional environment/atmosphere.

The third theme, “Challenges in Interdisciplinary Practice”, consists of three subthemes: facilitators, barriers, and suggestions for improvement.

**Figure 1:**
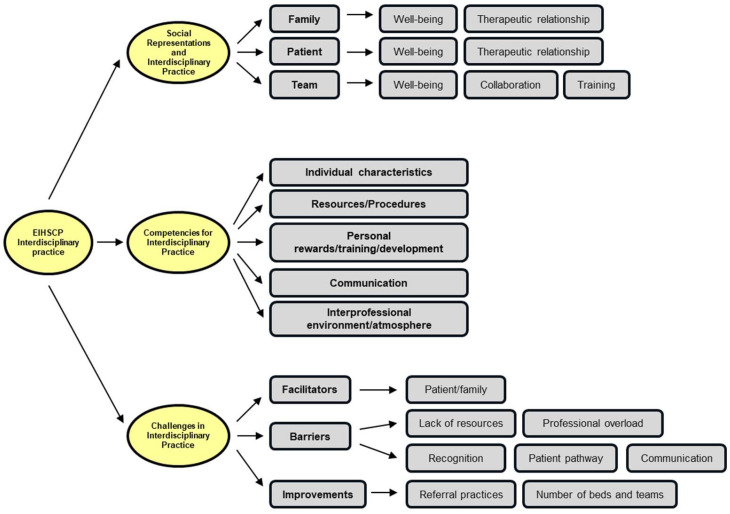
The coding tree of the thematic analysis on interdisciplinary practice in an EIHSCP.

Figure 1 provides a detailed overview of the organization of themes, subthemes, and corresponding codes.

#### 3.2.1. Social Representations and Interdisciplinary Practice

The participants’ responses highlighted key aspects related to the team, the patient, and, subsequently, the family/caregivers.

(a) Team

Regarding the interdisciplinary team, all professionals emphasized the importance of effective ‘team collaboration’ and the need to consistently consider the well-being of the team. This concern extends not only to internal dynamics but also to interactions with other hospital teams. Training was identified as a crucial factor, not only foundational and advanced training but also continuous education in PC, which enables the team to maintain a ‘holistic view of the disease’ in a unified manner.

(P9: Physician) *It means seeing various professional groups working together, where the sum of each one’s skills is greater than the individual parts (…) It is interesting and challenging to manage the boundaries between doctor and nurse (…) social worker, psychologist, and other necessary professions. Another role of the inter-hospital team within the hospital is to work in the wards, engaging with other professionals who receive support from the interdisciplinary team*.

(P10: Social worker) *The broad range of interdisciplinary knowledge enables more comprehensive care, promoting better quality of life, strengthening the professional-patient relationship, and reducing anxiety and stress*.

(b) Patient

The patient is a fundamental aspect of both the process and the interdisciplinary team. In this context, ensuring the patient’s well-being at all times is essential, supported by a therapeutic relationship that develops and strengthens over time. Ideally, this bond should extend beyond discharge, remaining accessible whenever the patient needs support, as recognized and expressed by them.

(P1: Physician) *The interdisciplinary approach, being comprehensive and holistic, addresses most of the needs of patients requiring palliative care (…) it provides some patients with a space for sharing and being heard, allowing for the assessment and prioritization of symptoms and the implementation of the most appropriate pharmacological and non-pharmacological therapeutic plan*.

(P7: Nurse) *The social aspect (…) the patient is discharged, and it is precisely important to also be able to communicate everything in the post-hospital discharge period*.

(c) Family

The family is also considered an integral part of the interdisciplinary approach, as highlighted by the participants and aligned with recommendations in the relevant literature. Equally important is the well-being of the family and communication with the family, which should be built on trust, clear and shared processes, and a collaborative effort toward a common goal: delivering excellence in PC.

(P6: Psychologist) *Collaboration among all is essential to ensuring the well-being of both the patient and their family, not in isolation, as a physician or psychologist alone, but through a comprehensive approach and effective team communication, which I believe is fundamental*.

(P10: Social worker) *The interdisciplinary approach in palliative care can have a positive impact on both the patient and their family, enhancing their overall well-being*.

(P11: Nurse) *Ongoing and active training is of utmost importance*.

The second theme focuses on Competencies for Interdisciplinary Practice.

#### 3.2.2. Competencies for Interdisciplinary Practice

(a) Individual characteristics

Participants explore various aspects, including experience, initiative, and interpersonal skills essential for teamwork. Interdisciplinary practice encompasses fundamental individual qualities such as compassion, humanity, empathy, respect for the patient’s dignity and autonomy, honesty, assertiveness, active listening, and ethical and cultural sensitivity. These personal attributes enable healthcare professionals to understand the vulnerabilities and needs of both the patient and their family, allowing them to act with compassion, respect, and ethical integrity. This approach facilitates decision-making and fosters the humanization of care, guided by bioethical principles: beneficence, non-maleficence, justice, and autonomy. Additionally, self-care and self-awareness are crucial considerations.

(P1: Physician) *Empathic capacity allows for appropriately adjusted compassionate attitudes without paternalism (…) Scientific rigor in both the working method and therapeutic strategies used (…) taking care of ourselves, knowing ourselves, and practicing self-care.*

(P11: Nurse) *Becoming aware of others’ suffering without judgment and with honesty (…) fostering attitudes that provide genuine support, ensuring that patients always feel they have a team that does not abandon them (…) a team that is present, available, and fully engaged*.

(P2: Nurse) *In effective interdisciplinary practice (…) addressing complex symptoms, guiding processes with ethical and cultural sensitivity, applying knowledge of emotional and spiritual support (…) and the ability to facilitate decision-making*.

(P4: Psychologist) *Maintaining compassion, humanity, and responsibility toward the patient, while acknowledging their concerns, vulnerabilities, and upholding our honesty*.

(P10: Social worker) *For effective interdisciplinary practice in palliative care, it is essential for the team to develop self-care and self-awareness skills. We can only care for others if we ourselves are well*.

(b) Resources and Procedures

The infrastructure and procedures must support the effective and efficient functioning of the team, a point emphasized by all participants. While physicians play a crucial role in symptom management, advanced care planning, and preparing families for possible disease progression scenarios, nurses are responsible for developing individualized care plans, managing symptoms, providing emotional and psychological support to patients and families, and coordinating care. Psychologists offer psychological support to both patients and their families, including bereavement care, while social workers assess socio-family needs, inform patients and families about available resources, and coordinate with the team, which may vary depending on each case’s specific needs.

Together, all professionals must base their practice on promoting well-being and quality of life for both patients and their families, following a person-centered approach. Several key processes were highlighted by professionals, including the development of individualized care plans, team meetings, family conferences, and the availability of resources both within the team and hospital and externally in the community.

Regarding human resources, professionals emphasized the importance of collaboration with dietetics and nutrition services as essential for individuals requiring PC, both in the hospital and in the post-discharge continuity of care at home. Additionally, the integration of information systems was highlighted as crucial to ensuring proper documentation and seamless communication across care transitions.

(P10: Social worker) *Interdisciplinary practices highlight the importance of family conferences, (…) the most relevant professionals for that situation should be present, or others should be called in, (…) for example, a nutritionist for a specific case*.

(P8: Social worker) *The approach in the first consultations, after assessing the needs, involves a socio-family diagnosis, engaging with the family and the patient, and explaining the available resources within the team (…) one of the professionals I immediately mention or inform as being available is the psychologist (…) seriously (…) it is an emotional trigger*.

(P4: Psychologist) *(…) it extends to the family, addressing concerns both in terms of what is happening and in the ongoing support and management of the patient (…) whether during hospitalization or solely through palliative care consultations (…) when the patient is at home and comes in for follow-ups. In bereavement support (…) which is a natural continuation and the final form of care we can offer to the family after the patient’s passing*.

(P2: Nurse) *Collaboration in the development of individualized care plans*.

(P11: Nurse) *Team meetings are also essential for jointly developing strategies (…) as well as (…) holding family conferences, coordinating care at different stages, aligning approaches, managing symptoms, providing emotional and psychological support, and establishing an advanced care plan*.

(c) Personal rewards/training/development

Personal rewards, ongoing training, and continuous professional development appear to enhance interdisciplinary work. Likewise, training and career development opportunities are seen as crucial, not only in terms of foundational education but also through advanced training and periodic courses on the subject. A continuous learning approach fosters both individual growth and collective progress within the team.

(P5: Nurse) *Theoretical training is highly important*.

(P3: Nurse) *Developing self-care and self-awareness skills*.

(P8: Social worker) *It is obvious that someone with basic training does not have the same level of knowledge as someone with a postgraduate degree. However, the basics provided me with a foundation to realize that I needed more detailed information and deeper knowledge. I believe that the more information is available, the more it facilitates practice*.

(P1: Physician) *It supports me in scientific rigor, the working methodology, and therapeutic strategies (…) Palliative medicine provides valuable guidance, along with continuous updates in pharmacology and other non-pharmacological approaches*.

(P12: Nurse) *Knowledge and skills are essential for the symptomatic management of total pain, as well as for providing support and information about community resources*.

(P8: Social worker) *I must say that when I was invited to take part in palliative care training, I approached it without any particular expectations. I went without fully knowing what to expect and wasn’t particularly motivated at first. However, this field turned out to be a pleasant surprise (…) I’m grateful to be here, knowing that, in some way, we are able to make a difference in the lives of individuals, or even many*.

(d) Communication

Effective communication skills within the team, supported by appropriate systems and strategies, are a decisive factor in fostering collaboration, guiding decision-making, and ensuring efficient teamwork. Communication is essential, not only among team members but also with patients and their families. In a complex environment such as the hospital setting, strong communication facilitates interaction and enhances interdisciplinary teamwork. The ability to communicate calmly and assertively stands out as a crucial skill for the optimal functioning of interdisciplinary teams.

(P2: Nurse) *The ability to communicate effectively, work as a team, and collaborate with other healthcare professionals plays a key role in addressing complex symptoms*.

(P1: Physician) *Agile and assertive communication is crucial, free from paternalism (…) ensuring high-quality interactions within the team (…) and meaningful communication with patients and caregivers*.

(P6: Psychologist) *Communication is essential in this type of work and intervention (…) whether with colleagues, families, or patients, as it can sometimes become complex and delicate*.

(P12: Nurse) *Empathy, sincerity, adapted communication, and ethics*.

(e) Interprofessional environment/atmosphere

A team culture built on trust, the recognition of each member’s contributions, and an approach that fosters consensus and interprofessional collaboration appears to be a key factor in highly competent interdisciplinary teams. This environment prioritizes both the patient and their family. This theme, although less frequently mentioned than others, was primarily identified by participants with over six years of experience and advanced training in PC, suggesting a deeper perception associated with their specialized expertise.

(P10: Social worker) *Being able to put ourselves in another’s place, recognizing their suffering without judgment, and honestly fostering attitudes that provide meaningful support during this stage of their life, while ensuring they always feel surrounded by a team that is present, engaged, and never abandons them*.

(P6: Psychologist) *Beyond inpatient care and the support provided to hospitalized patients, the family is also included in this process. There is always a concern for their well-being, both in understanding what is happening and in fostering a climate of mutual support*.

(P1: Physician) *There must be a commitment to ethical conduct with a high level of maturity*.

(P8: Social worker) *Being a truly interdisciplinary team and trusting one another are serious challenges in the fast-paced environment of a hospital setting*.

The third theme focuses on Challenges in Interdisciplinary Practice and is organized into three subthemes: (1) facilitators, (2) barriers, and (3) suggestions for improvement.

#### 3.2.3. Challenges in Interdisciplinary Practice

(a) Facilitators

Among the facilitating factors mentioned by the team is the inclusion of the patient and family within the team itself. Interdisciplinary healthcare teams face a specific set of challenges that are not typically found in other types of teams. This is particularly evident in PC teams, where coordinated action among all members, including the patient and their family, is recognized as a key facilitating element. These teams inherently encounter challenges related to the often contentious nature of shared professional roles and knowledge, collaborative planning, and joint decision-making. Nevertheless, they are committed to delivering high-quality care to patients in complex settings, such as the hospital environment (Hospital Centers). As some participants stated the following:

(P8: Social worker) *I believe we need to have enough humanity to adapt to other professionals, because it’s not just about each person’s profession, obviously, everyone has their own approach. We’re also talking about professionals’ personal beliefs, their temperament, and the degree of openness each one brings (…) it’s about adapting to one another’s temperament and working style, so that the team can function as seamlessly as possible, always in service of the patient*.

(P3: Nurse) *Well-structured teams (…) are characterized by mutual respect among team members*.

(P2: Nurse) *It greatly facilitates the active participation of patients and their families*.

(b) Barriers

The main barriers identified were as follows: lack of resources; professional stress and overload; and lack of recognition from institutions and peers.

The lack of resources refers not only to the types of professionals available but also to the amount of time they are able to dedicate to the teams, particularly those who are not part of the core team outlined in the National Strategic Plan, such as nutritionists, physiotherapists, occupational therapists, and others. Professionals in therapy and rehabilitation were not addressed in this study. On this topic, the following sentiment was repeatedly expressed:

(P2: Nurse) *Resources are lacking*.

(P9: Physician) *Unfortunately, the barrier that everyone remembers is the insufficient availability of resources, both in terms of quantity and dedicated time. We still encounter situations where teams only have a psychologist for 10 h a week (…) and the same goes for social workers. It doesn’t work, even if those professionals are highly competent*.

Clearly, the previously mentioned challenge is closely tied to another: professional stress and overload. Several insights were shared on this matter, including the following:

(P2: Nurse) *In terms of barriers, the emotional exhaustion of palliative care professionals is highlighted, as well as the absence of recognition by professionals from other areas*.

(P9: Physician) *The professionals work under too much stress, under emotional overload (…) they are professionals who are permanently at risk of developing… well, situations of despair and so on*.

(P1: Physician) *Recognition of the specialty by hierarchical superiors in administration and the Ministry of Health, as well as by civil society. Recognition of the emotional and moral exhaustion of professionals in this field (…) Inadequate patient-to-healthcare professional ratios*.

As reported in the literature, other challenges were also identified, particularly regarding the recognition of professionals and institutions. Institutions tend to engage less, or less consistently, with PC teams, possibly due to a lack of understanding about what it means for a patient or family to have PC needs. This barrier can compromise the quality of care and hinder the entire process of referral and integration across the different levels required within the patient care pathway.

(P2: Nurse) *Stigma associated with palliative care*.

(P3: Nurse) *Difficulty in demystifying who palliative care is intended for*.

(P1: Physician) *Recognition of the specialty by hierarchical superiors of the administration and the Ministry of Health, as well as by civil society*.

Other minor barriers, as mentioned by nursing professionals, include issues related to the patient care pathway and communication, particularly outside the hospital setting. On this topic, these were the testimonies noted:

(P3: Nurse) *There is a lack of community-based services to ensure continuity of care for these patients and their families (…) which is crucial for the patient care pathway*.

(P2: Nurse) *Differences in values, perspectives, as well as cultural and linguistic barriers*.

(P3: Nurse) *Difficulty in communication with other teams*.

(c) Improvements

Finally, participants offered several suggestions for improving interdisciplinary practice in PC. These suggestions focused on best practices in referral processes, the importance of knowledge and research, and the need to increase the number of beds and specialized teams. In this regard, improvements in referral practices, enhanced knowledge and research, and the expansion of care capacity appear to be particularly relevant. A few key statements illustrate these points:

(P5: Nurse) *Good practices to ensure timely referral (before the patient is admitted to the team and during discharge planning)*.

(P5: Nurse): *The need to share and disseminate the core principles of palliative care*.

(P9: Physician) *There are many people experiencing collective anxieties within the general population. People are in the hospital, at home, wherever they choose (…) with the right to make that choice.*

(P1: Physician) *It is essential to promote health literacy in the field of palliative care (…) and to promote meaningful research in this area*.

(P4: Psychologist) *We have six temporary inpatient beds (…) which is not enough to meet the demand and needs of the population*.

(P9: Physician) *As long as a response is available, which is itself another limitation, the coordinating role of teams, both internally and with others, working together, is not always easy (…) it relies on highly intensive communication among all parties involved*.

## 4. Discussion

This qualitative study provided an in-depth exploration of perceptions and experiences related to interdisciplinary practice within an EIHSCP. Continuous training emerged as a key factor in ensuring the effectiveness of interdisciplinary work in PC. Notably, eight out of the twelve participants had advanced training in PC, while ten had a foundational education in PC. This team composition significantly influenced their expertise and perspectives on the subject under investigation [2,3,5,6,7,10,13,14,18,19].

Based on their occupations and professional roles within the team, it is evident that physicians play an active role in service management, medication oversight, and patient consultations, as well as in planning discharges and admissions. They also have a central coordinating function, facilitating communication among all professional areas and with hospital services beyond the Inpatient Palliative Care Team, particularly in collaboration with the nutrition department.

Nurses are responsible for pain management and the administration of medications. They coordinate with families regarding care during hospitalization and prepare them for discharge and post-discharge care. Nurses also oversee the management of patient records and participate in case discussions and family conferences. Additionally, they play a key role in organizing training and providing consultation to other hospital departments.

Social workers and psychologists are essential in supporting both patients and their families. They regularly participate in family conferences, offer grief counseling, and provide emotional and psychosocial support both before and after death. Furthermore, they contribute significantly to team support and are responsible for managing interactions with community partners and other hospital services.

The main facilitator of interdisciplinary work within the EIHSCP in this highly complex context is the team’s structured involvement of both the patient and their family, an approach that aligns with the national and international literature on best practices in PC. It appears that even professionals with only one year of experience and without advanced training in PC view this as a critical factor [1,2,3,8,15,19].

As for the barriers, professionals identified several external factors beyond the direct control of the team. These include a lack of resources, professional stress and overload, a limited recognition of professionals and institutions, care pathway inefficiencies, and challenges in team communication, particularly with other hospital services and external providers. These are clearly escalating challenges for healthcare teams, especially as the population continues to age, placing increasing pressure on health services in general and on PC services in particular [4,5,6,7,8,9,10].

Regarding the social representation and interdisciplinary practice, it was evident that participants’ focus, based on their responses, centers primarily on the team, followed by the patient, and then the family or caregivers. This is unsurprising, given that the context of an EIHSCP within a hospital setting naturally shapes these priorities. In such environments, access to families can be more limited and often occurs at prearranged times. The patient, being in a temporary inpatient setting and under the continuous care of the team, remains the central focus 24 h a day. Admissions are short-term and limited to the period necessary for symptom control, clinical stabilization, or to provide support during the final stage of life, prior to discharge back into the community [33,36].

Consistent with the literature, collaboration among professionals contributes significantly to the well-being of both patients and their families, ensuring high-quality care for all involved, core elements of interdisciplinary PC. The patient’s well-being must be consistently supported through a strong therapeutic relationship, which should be carefully planned, long-term, and extend well beyond hospital discharge [2,3,5,6,7,10,13,14,15,16,17,18,19].

Regarding the competencies for interdisciplinary practice within hospital-based PC teams, the findings suggest that these go well beyond the professionals’ technical and scientific expertise. The data confirm that health teams engaged in interdisciplinary PC face a specific set of challenges that are not typically encountered by other types of healthcare teams. These challenges arise while delivering high-quality care to complex patients within a highly demanding and intricate environment, such as that of a hospital setting, particularly within a hospital center [3,20,27,34].

Among the core competencies identified in the literature as fundamental to interdisciplinary teamwork in PC, all participants placed individual characteristics at the forefront, attributes to be demonstrated in the daily practice of interdisciplinary care within an EIHSCP. This aspect was particularly emphasized by those with more years of experience and advanced training in PC, who highlighted the critical role of each professional at the micro level, while maintaining alignment with the institution’s strategic goals at the meso level. The importance of possessing knowledge, experience, initiative, self-awareness regarding both strengths and weaknesses, active listening skills, and a capacity for reflective practice, as well as a genuine willingness to work toward shared goals, was strongly validated. Participants also spoke about self-care and self-knowledge as essential elements [3,34].

In addition, they significantly emphasized the role of procedures and processes, reinforcing the need for adequate systems and infrastructure to support the team’s vision. Examples included regular team meetings, a conducive work environment, integrated communication systems, appropriate referral criteria, and family conferences [1,3,34].

Another important area of competency relates to personal rewards, training, and professional development. The findings confirmed that having advanced training in PC leads to a greater awareness of and value placed on ongoing education and development over time. Professionals with more experience and advanced training consistently emphasized the need for structured, systematic education, while those with less experience and no advanced training either did not mention the topic or addressed it only superficially. This underscores the importance of giving structural and institutional weight to education, not only in initial training but also in lifelong learning and workplace-based education. Career development, the enrichment of professional CVs, as well as the availability of rewards and opportunities that foster both individual and collective motivation, were seen as essential. In short, the presence or absence of advanced training in PC strongly influences how professionals perceive and value long-term education and development [1,3,34].

Communication was another widely recognized competency. This aligns with the literature, which identifies communication as foundational to interdisciplinary work in PC. Assertive, empathetic, and context-sensitive communication strategies enhance collaborative decision-making and improve responses to complex symptoms. Regarding the interprofessional atmosphere, participants validated the importance of a work environment grounded in trust, collaboration, and the appreciation of all professionals as essential for effective interdisciplinary practice. Fostering a climate of mutual support and respect benefits not only the team but also the patients and their families [2,3,4,12,16,17,20,29,33,36].

In the hospital setting, where challenges are constant, clear, non-paternalistic communication was seen as strengthening the therapeutic relationship and optimizing care delivery. Ethical maturity and professional cohesion were also viewed as especially relevant in such demanding environments, ensuring comprehensive and humanized care [2,3,4,12,16,17,20,29,33,36].

Based on the participants’ input, several suggestions for improving interdisciplinary practices in hospital-based PC teams emerged. These included the implementation of best practices in referral processes, increased knowledge and research in the field, and the potential expansion of both the number of beds and the number of dedicated teams. These suggestions point to systemic issues that could be revisited during a future review of the current organizational model for PC delivery within the SNS, which still requires structural improvements across different levels of the system to yield tangible results at the micro level, namely, in the care delivered by frontline teams.

Participants also highlighted the need for a more strategic and integrated approach to PC within the Portuguese healthcare system. The integration of care should be rethought at all levels, ensuring that the patient is not seen merely as a recipient of care but as an active participant in a continuous and coordinated care journey in Portugal [2,3,4,13,15,16,17,29,30].

### 4.1. Strengths and Limitations

Based on the available findings, several strengths and limitations can be identified.

One of the major strengths of this study was the opportunity to interview all members of the EIHSCP in the Médio Tejo region. However, this strength is also tied to a limitation, as the specific reality of this team may not reflect the experiences of other teams at the regional or national level. This highlights the need for further research involving EIHSCPs across the country, including those in the autonomous regions of the Azores and Madeira.

Among the study’s strengths is the confirmation that interdisciplinary collaboration enables the type of holistic approach envisioned in PC, one that includes and prioritizes both the patient and their family. The findings highlight that the team functions effectively regardless of individual members’ prior service experience or length of time working specifically in PC. A notable strength is the team’s inclusive dynamic, wherein more experienced professionals actively support and mentor those with less experience.

It would also be valuable to conduct studies that incorporate the perspectives of patients and families, as their involvement in interdisciplinary practice is clearly recognized by the team. While the integration of patients and families into interdisciplinary work appears to be well established, it is essential to gather their perceptions on this process.

Furthermore, what happens beyond the team, particularly in terms of care transitions and discharge pathways, also warrants deeper investigation. Participants expressed concern about the continuity of interdisciplinary care beyond the hospital setting, particularly regarding integration with other services and community-based care. This reinforces the need to examine how interdisciplinary practices are maintained across different levels and phases of care.

Regarding limitations, while the core team functions effectively, a shortage of both material and human resources compromises the efficiency and quality of care. This scarcity contributes to professional overload and stress, and it poses significant challenges to inter-institutional communication. Communication between hospital-based teams and external services remains problematic, undermining the continuity of care. These are critical areas that require further reflection and improvement.

Moreover, a persistent lack of understanding about the true scope of PC continues to hinder the development of interdisciplinary competencies within healthcare organizations. The current referral and integration model for PC within the SNS is inadequate and calls for structural reforms. Such improvements must address organizational frameworks, resource allocation, and policy development to enable a more effective, coordinated, and genuinely patient- and family-centered approach. Optimizing care delivery is essential to ensuring accessible, continuous, and comprehensive support for patients and their families.

### 4.2. Practical Implications

Based on the findings of this study, several practical implications can be identified, both for settings where an Inpatient Palliative Care Support Team (IPCS Team) is already established and for those where such a structure is yet to be implemented.

First, the importance of fostering regular collaborative dynamics is underscored through the consistent use of interdisciplinary meetings and the co-development of care plans. These practices promote the alignment of interventions, facilitate knowledge exchange, and enhance team cohesion.

In addition, the creation of support tools, such as shared digital platforms and integrated care protocols, can significantly improve communication, support clinical decision-making, and ensure the continuity of care, especially in contexts where time and resources are constrained.

Organizational culture and leadership also emerge as critical factors. Cultivating a culture grounded in mutual respect and interprofessional support, alongside the development of inclusive leadership that ensures equity and encourages active engagement from all team members, is essential.

Professional training is another key component in this process. It should aim to deepen the understanding of the value of interdisciplinarity, not only within the team but also in extending this perspective to the broader care network. There is a clear and growing need to invest in high-quality, continuous, and advanced training that helps standardize knowledge across professionals. This type of education is vital for maintaining the quality of care and reinforcing the collective commitment to interdisciplinary practices. Emphasis should also be placed on training in assertive communication and fostering a culture of mutual support, as these elements are foundational to effective interdisciplinary work environments. Enhanced awareness and alignment with the practical realities of implementing palliative care also contribute to better team functionality and cohesion. Rethinking the structure of continuing education programs to explicitly incorporate content on interdisciplinarity in palliative care would be a valuable step forward.

Lastly, it is recommended that patients and their families be systematically included as active members of the interdisciplinary team. This person-centered approach promotes shared decision-making and ensures that care delivery aligns with patients’ individual values and preferences.

These recommendations are broadly applicable and may significantly enhance interdisciplinary practice, even in healthcare settings where formalized structures such as the IPCS Team are not yet in place.

## 5. Conclusions

This study is likely one of the first in Portugal to explore the perceptions and practices of interdisciplinary care within hospital-based PC teams, which play a crucial role at the transition between inpatient services and community-based care. Ensuring a greater awareness and integration of PC in this setting is essential for achieving a meaningful and lasting impact.

Despite the dedication of teams and the emphasis on continuous training, structural and organizational challenges still limit the effectiveness of interdisciplinary hospital-based PC teams. These challenges are particularly evident when coordination is required beyond the core mandated professions, extending to other hospital services and community-based teams.

Interdisciplinary collaboration is most tangible in teams that prioritize patient- and family-centered care, although this approach also presents significant challenges. A positive interprofessional environment, built on trust, collaboration, and mutual respect, plays a key role in sustaining effective practice. Additionally, ongoing professional development is essential for fostering clear communication, ethical commitment, and cultural sensitivity. While there is still room for growth, critical factors, such as structured team meetings, family conferences, efficient communication systems, and well-defined referral criteria, were identified as fundamental.

In summary, strengthening interdisciplinary PC requires improving resources, enhancing communication, and refining the organizational model to ensure a more integrated and effective approach.

## Figures and Tables

**Table 1:** Sociodemographic characterization of the sample.

Participant	Age	Gender	Profession	Professional Experience (Years)	Professional Experience in PC (Years)	Advanced Training in PC	Other PC Training
P1	41	Male	Physician	15	7	Yes	No
P2	30	Female	Nurse	15	6	No	Yes
P3	41	Female	Nurse	15	9	Yes	Yes
P4	36	Female	Psychologist	11	6	Yes	Yes
P5	38	Female	Nurse	15	9	Yes	Yes
P6	28	Female	Psychologist	1	1	No	Yes
P7	26	Female	Nurse	5	2	No	No
P8	47	Female	Social worker	17	14	No	Yes
P9	52	Male	Physician	30	23	Yes	Yes
P10	39	Male	Social worker	15	13	Yes	Yes
P11	41	Female	Nurse	15	14	Yes	Yes
P12	47	Female	Nurse	17	7	Yes	Yes

## Data Availability

The raw data supporting the conclusions of this article will be made available by the authors on request.

## Abbreviations

The following abbreviations are used in this manuscript:

PC	Palliative care
EIHSCPs	In-Hospital Palliative Care Support Teams
SNS	National Health System (*Serviço Nacional de Saúde* in Portuguese)

## Appendix A. Interview Guide

Q1: What does interdisciplinary practice in palliative care within a hospital-based interdisciplinary team mean to you?
Q2: In what ways does training in palliative care contribute to an interdisciplinary practice centered on the needs of patients and families within a hospital-based interdisciplinary team?
Q3: How does interdisciplinary practice in palliative care influence the person receiving care, as well as the professional–patient relationship? Could you provide some examples?
Q4: What knowledge and skills do you consider essential for effective interdisciplinary practice in palliative care?
Q5: What interdisciplinary practices or activities do you engage in as part of your professional routine? Could you give some examples?
Q6: What attitudes and values do you believe are necessary for interdisciplinary work in palliative care? Can you share any examples?
Q7: What barriers do you identify to interdisciplinary practice in palliative care?
Q8: What facilitators do you recognize in supporting interdisciplinary practice in palliative care?
Q9: Is there anything else you would like to add regarding this topic?
